# Barriers in phase I cancer clinical trials referrals and enrollment: five-year experience at the Princess Margaret Hospital

**DOI:** 10.1186/1471-2407-6-263

**Published:** 2006-11-08

**Authors:** Jeremy Ho, Gregory R Pond, Colin Newman, Martha Maclean, Eric X Chen, Amit M Oza, Lillian L Siu

**Affiliations:** 1 Department of Medical Oncology and Hematology, Princess Margaret Hospital, University Health Network, 610 University Avenue, Toronto, Ontario M5G 2M9, Canada; 2 Department of Clinical Study Coordination and Biostatistics, Princess Margaret Hospital, University Health Network, 610 University Avenue, Toronto, Ontario M5G 2M9, Canada

## Abstract

**Background:**

There is a paucity of literature on the referral outcome of patients seen in phase I trial clinics in academic oncology centres. This study aims to provide information on the accrual rate and to identify obstacles in the recruitment process.

**Methods:**

A retrospective chart review was performed for all new patients referred and seen in the phase I clinic at the Princess Margaret Hospital between January 2000 and June 2005. Data on their demographics, medical history, and details of trial participation or non-entry were recorded.

**Results:**

A total of 667 new phase I referrals were seen during the stated period. Of these patients, 197 (29.5%) patients were enrolled into a phase I trial, and 64.5% of them started trial within 1 month of the initial visit. About a quarter (165 of 667) of the patients referred were deemed ineligible at their first visit, with the most frequent reasons for ineligibility being poor performance status, unacceptable bloodwork, too many prior treatments and rapid disease progression. The remaining 305 patients (45.7%) were potentially eligible at their initial visit, but never entered a phase I trial. The main reasons for their non-entry were patient refusal, other treatment recommended first, and lack of available trials or trial spots.

**Conclusion:**

This study provides information on the clinical realities underlying a referral to a phase I clinic and eventual trial enrollment. Better selection of patients, appropriate education of referring physicians, and opening phase I trials with fewer restrictions on some criteria such as prior therapy may enhance their recruitment rates.

## Background

Phase I clinical trials evaluate the dosing and toxicities of novel agents or combinations of agents in humans after appropriate preclinical testing of safety, toxicology and pharmacology. Oncology patients who have been referred to the phase I trials clinic represent the unique subset of patients who have exhausted standard treatment options, yet continue to be functionally well. Because the risks associated with an investigational drug are unknown, and the likelihood of therapeutic response is relatively small [1], multiple studies have examined the motivations of these patients for participating in phase I oncology trials. While patients understand that it is not the purpose of phase I studies, the potential for receiving personal therapeutic benefit remains the most important motivator for trial participation [2-5]. However, in a recent review of over 10,000 participants in phase I trials sponsored by the National Cancer Institute between 1991 and 2002, the average response rate for all agents was 10.6%, and agents given in first-in-human trials produced a response rate of only 4.8% [6].

Although clinical trials are a crucial step in the development of new cancer treatments, their progress can be limited by low accrual rates. Barriers to the enrollment of newly diagnosed cancer patients into phase I to III oncology trials have been identified primarily as patient ineligibility, lack of trials, and patient refusal [7,8]. However, due to the distinctive nature of phase I clinical trials and its patient population, the obstacles facing recruitment into phase I studies are likely to be different than those observed in oncology trials as a whole. Unfortunately, little data exist to address this issue, even though clarification of these obstacles could aid in improving and streamlining the referral and evaluation process in phase I oncology trials. Therefore, to address this question, we retrospectively reviewed the course of patients from the point of referral to trial entry or non-entry at the Princess Margaret Hospital (PMH) phase I trials clinic over the past 5 years.

## Methods

### Chart Review

PMH is a tertiary care cancer center in Toronto, Canada, with a well established phase I clinical trials program since the late 1990s. All new patients referred to the phase I clinic from January 2000 to June 2005 were identified through clinic lists. Paper and/or electronic medical records from the initial clinic visit to the time of trial entry or non-entry were reviewed. Demographic data (gender, age, postal code), medical information (tumor site, number of prior chemotherapy regimens, performance status, referring physician), details of trial participation (trial entry date, trial entered, reasons for delayed entry into trial), and/or circumstances around trial non-entry were abstracted from physicians' clinical notes that were dictated after each clinic visit. A delay in trial entry was arbitrarily defined as greater than 1 month from the date of the initial clinic visit to the date of starting study drug(s).

Reasons for trial non-entry were mutually exclusive, whereas the circumstances surrounding these reasons could be multiple and were not mutually exclusive. The reasons for trial non-entry were ranked in a hierarchical order determined by consensus of the authors: refusal; doing well and hence no trial treatment recommended; no trials or spots available; other treatment recommended first; initially a candidate but deteriorated while waiting; lost to follow-up or reasons for non-entry unknown. The reasons were ordered from the most to least limiting factor in patient accrual, as well as the clinical flow of a patient's assessment in the phase I clinic. For instance, if a newly referred patient with painful bony metastases declined participation after the initial consultation, and the phase I physician would have recommended palliative radiotherapy before enrollment into any phase I trials, the patient's refusal would be recorded as the only reason for trial non-entry. Among 305 patients who were potentially eligible but ultimately did not enter into a phase I trial, only 73 (24%) had 2 or more reasons for trial non-entry in this retrospective chart review.

All data abstracted were entered into a password-protected database. This retrospective chart review study was approved by the Research Ethics Board at the PMH, University Health Network. This project is internally funded at the Princess Margaret Hospital and there are no external funding sources.

### Statistical and Study Analysis

Descriptive statistics, such as the mean, median, inter-quartile range [IQR], standard deviation [sd] and proportion were used to summarize patient characteristics and outcomes. Distance to cancer centre in kilometers was calculated as linear distance from the patients' listed residence to PMH via the forward sorting area (FSA). The FSA is denoted by the first 3 alpha-numeric digits in the Canadian postal code. The mid-point for each FSA was obtained by using the postal code conversion file (Postal code conversion file January 1999 edition, produced by Statistics Canada) [9], obtained from the data library service of the University of Toronto, Toronto, Canada. Patients were assumed to reside at this central point of their respective FSA.

## Results

### Patient Characteristics

Six hundred and sixty-seven patients were identified as new referrals to the phase I oncology clinic. Patient demographics are summarized in Table 1. Interestingly, 68 (10.2%) chemonaïve patients were referred to the clinic. These appeared to represent patients with malignancies without effective standard treatments, or patients who were considered for phase I trials that contained at least one active agent in combination with novel agents.

### Referral Outcomes

**Figure 1:**
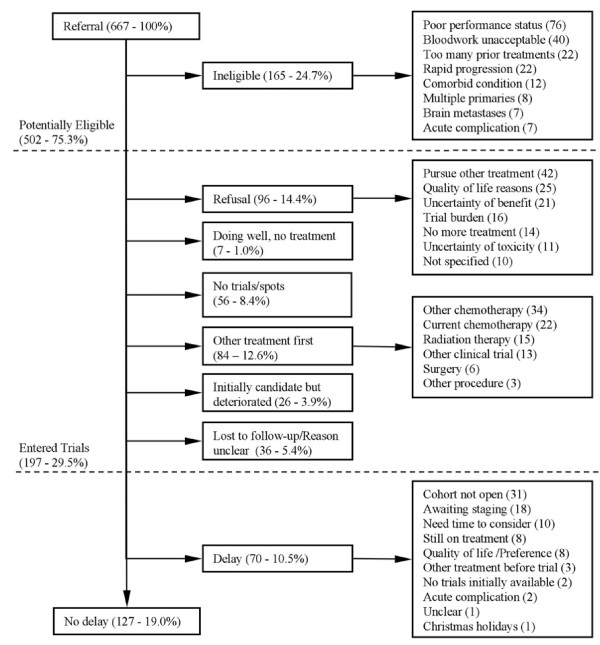
Patient outcome from the point of referral.

The outcome of the patients from the point of referral is summarized in Figure 1. Of the 667 new patients, 165 (24.7%) were ineligible for trial participation, the most common reasons being poor performance status in 46% (76), unacceptable bloodwork in 24% (40), too many prior treatments in 13% (22), and rapidly progressive disease in 13% (22).

Out of the total of 667 patients, there were 96 (14.4%) patients who declined participation in a phase I trial. The circumstances given for declining participation were a desire to pursue other treatments (n = 42; 44%), quality of life reasons (n = 25; 26%), uncertainty of benefit (n = 21; 22%), trial burden (n = 16; 17%), not wanting further treatment (n = 14; 15%) and uncertainty of toxicity (n = 11; 11%). Just over half (n = 23; 55%) of the 42 patients who wished to pursue other treatments went on to receive conventional treatments, but 12 (29%) received alternative treatments, and 7 (17%) went on another clinical trial.

Besides patients' refusal to take part in phase I trials, the other reasons for potentially eligible patients for not being enrolled were as follows. Of the 667 patients, 7 patients (1.0%) were completely asymptomatic and as such, entry into a trial was not recommended at the time. No trials or available spots were available for 56 patients (8.4%), and other treatments were recommended prior to a phase I trial for 84 (12.6%) patients. Twenty-six patients (3.9%) were in the process of being worked-up for a phase I trial, but were unable to enroll due to deterioration in their clinical conditions. Thirty-six patients (5.4%) were lost to follow-up or it was unclear why they did not enter a trial.

Ultimately, 197 of 667 patients (29.5%) were entered into a phase I trial. When analyzed by the year of accrual, the proportions of patients who entered into a phase I trial did not differ significantly over time. The enrollment percentages were 36% in 2000, 29% in 2001, 26% in 2002, 25% in 2003, 31% in 2004 and 29% in 2005. The majority (127 of 197; 64.5%) of patients entered on a phase I trial did so within 1 month of the initial clinic visit, with a median time to trial entry of 2.4 weeks. Seventy of 197 patients (35.5%) experienced a delay in entering a trial, with a median time to trial entry of 7.1 weeks. Common reasons for a delayed trial entry included awaiting trial cohorts to open (n = 31; 44%), waiting for trial work-up (n = 18; 26%), needing time to consider (n = 10; 14%), being on another treatment (n = 8; 11%) and patient preference (n = 8; 11%). Only 2 (3%) patients were delayed because of lack of immediately available trials.

### Subgroup Analysis

A breakdown of patient outcomes by patient characteristics is shown in Table 2. Neither gender nor distance appeared to affect patient outcome. However, age did appear to be a factor as a greater proportion of ineligible patients were over 70 years of age and patients less than 70 were more likely to be entered on trial. Tumor site was also an important factor in affecting trial entry outcomes. Tumor sites that were least likely to enter patients into phase I trials included sarcoma, lung and breast cancers. Ineligibility appeared to be the most prevalent reason for non-entry in breast cancer and sarcoma patients. Lung cancer patients appeared to be eligible for phase I trials at initial consultation visits, but many were recommended other treatments first, or had rapid deterioration after initially being eligible. Patients with multiple primary cancers were always excluded from trial entry. Additionally, heavily pre-treated patients (3 or more previous chemotherapy regimens) and those with poor performance status at the initial clinic visit (ECOG 2 or higher) were more likely to be ineligible. Patients who had been referred to the phase I trial clinic by oncologists at PMH were more likely to go on trial and less likely to be ineligible when compared to patients referred by external oncologists.

## Discussion

This paper represents the first study to examine the referral and enrollment process in the phase I trial setting. A previous study by Corrie et al. reviewed the factors limiting the recruitment of 1,411 patients into phase I to III oncology trials at the West Anglia cancer research network in the United Kingdom [7]. Although their overall recruitment rate was 19%, no trials were available for 40% of patients, 32% of patients were ineligible, and 19% of eligible patients declined entry into a study. Another study by Lara et al. at the University of California Davis Cancer Center in the United States prospectively assessed patient accrual into phase I to III oncology trials and reported an accrual rate of 14% out of 276 patients [8]. Thirty-seven patients, or 49% of those they considered eligible for available protocols, refused to participate for reasons including a desire for other treatment, distance from the cancer center, and insurance denial. While Lara et al. described financial barriers to trial participation, these obstacles were not faced by our patients due to the universal healthcare access provided by the Canadian medical system. However, recent legislation in certain states such as California, Georgia, and Massachusetts, ensures insurance coverage for phase I clinical trials, making them more accessible to patients in those states [10]. Thus, our results should be generalizable to large academic centers with phase I programs outside of Canada.

In our review of 667 new referrals to the phase I clinic at PMH from 2000 to 2005, the overall accrual rate was 29.5%. While not directly comparable to those studies examining recruitment to all three phases of clinical trials, our accrual rate is substantially higher. The higher accrual rate seen in our study likely reflects the fact that patients were referred to our dedicated phase I clinic with the expressed intent of participating in a phase I trial. However, 24.7% of patients were still ineligible, 14% declined to enter a trial, 12.6% were recommended another treatment prior to a clinical trial, and 8.4% were unable to participate due to a lack of available trials or open cohorts. These results suggest that patient recruitment could be further improved by reducing the impact of several of these factors.

Often characterized as desperate and willing to undergo any treatment for a small chance of benefit, it seems surprising that 14% of newly referred patients, or 19% of eligible patients, declined participation in a phase I trial. However, this is similar to the rate reported by Corrie et al. [7], and lower than that reported by Lara et al [8]. Patients had multiple reasons in deciding not to participate, the most common of which was the desire to pursue other treatments, similar to the finding of Lara et al [8]. However, other important reasons included quality of life purposes, the uncertainty of benefit and toxicity, trial burden, and the desire to have no more active treatments. This finding suggests that many phase I patients do comprehend the requirements and objectives of study participation. Although phase I trial physicians are the best qualified to help patients make this decision, educating patients about the basics of phase I trials prior to their referral may help some patients determine then that it is something they do not wish to pursue. This would, in turn, make more spots available for eligible and interested patients, as well as shorten the waitlist to be seen in phase I clinic.

Although it is usually thought that patients are referred for phase I trials only after exhausting all other treatment options, a remarkable 12.6% of newly referred patients, or 16.7% of eligible patients, were recommended another treatment prior to a phase I study. Clinical experience shows that trial physicians may make such a suggestion if they feel that another treatment is better at providing symptomatic control, or can provide more benefit than the 5 to 10% response rate obtained from an experimental agent. Most of these patients are informed that they can be reevaluated for a phase I trial after they complete the recommended treatment, but unfortunately, the vast majority of them are unable to do so. Because the clinical course of these end-stage patients is unpredictable, the window of opportunity for starting a phase I trial may be limited. The delay involved in waiting for an appointment, being assessed in phase I clinic and then undergoing another therapy may be sufficient time for a patient's clinical status to deteriorate, thus rendering the patient ineligible for trial entry. Therefore, in order to expedite patient accrual into trials, it is important to ensure that all treatments have been exhausted and all symptoms are well managed prior to referral to the phase I clinic. Interestingly, 47% of chemonaïve patients in our study entered into phase I trials (data not shown), and were less likely to be recommended another treatment first (15%). These figures indicate that these were indeed the patients with malignancies for which no effective standard treatments exist, or alternatively these patients were accrued into phase I trials containing at least one active approved anticancer agent.

Lack of trial availability or open cohorts prevented 8.4% of newly referred, eligible and interested patients in our study from participating in a trial. Additionally, it was the most common reason for a delay in patients who eventually entered a trial. Given that trials were not available for 40 to 50% of patients in other studies [7,8], our result is intriguing, although the phase I trial setting is different in that trials are neither site nor stage specific, allowing patients to enter into any open trials. Also, in contrast to the other studies which reviewed experiences in general cancer clinics, patients in our study were seen in a dedicated phase I clinic in which enrollment into a phase I trial is the main reason for the referral, and the physician and nursing staff are well aware of trial availability. Nonetheless, if more trials and spots were made available, all eligible and interested patients could be accommodated and the amount of delay in starting a trial reduced.

While nearly one quarter of newly referred patients were deemed ineligible to enter a phase I trial for various reasons, the subgroup analysis also identified specific patient factors that were more likely to preclude trial entry. Elderly patients 70 years of age or over appeared less likely to be eligible for and enter phase I trials when compared to patients less than 70 years old. The finding that elderly patients are underrepresented in oncology trials, including phase I studies, has been previously documented [11,12]. Tumor site also affected clinical outcome, as breast cancer patients in particular were more likely to be ineligible. A further review of the data indicates that 72% (13/18) of the ineligible breast patients were excluded from trial entry because they had received too much prior treatment. The number of sarcoma patients in this study was too small to draw any conclusions about their reasons for ineligibility. Certainly, those with multiple primary cancers were always excluded from phase I trials and therefore should not be referred to the clinic for assessment. Additionally, patients with poor performance status (ECOG >2) were very unlikely to enter a trial, which is further supported by our finding that this was the most common reason for ineligibility. Heavily pre-treated patients were also unlikely to enter a trial, and accounted for 13% of patients who were excluded by trial entry criteria. Although there exist trials without restrictions on the number of prior lines of chemotherapy, this obstacle could be overcome by increasing the number of these trials. Especially in the current era of targeted agents where bone marrow toxicity is less common, the amount of prior treatment is less of a concern. Furthermore, trials with unrestricted prior therapy are generally easier to complete. Although a relaxation of some trial eligibility criteria could increase accrual rates, trial investigators would need to balance this with clinical or scientific concerns, such as avoiding undue toxicity or maintaining a more homogenous patient group.

Finally, being referred by an external physician appeared to decrease a patient's chances of entering a trial as more of these patients were ineligible or were recommended another treatment first. When compared to patients referred by PMH physicians, those referred by external physicians were more likely to have an ECOG status of 3 (4.6% vs. 10.7%) and thus be excluded from trial entry due to poor performance status (40% vs. 51.7%). PMH physicians may have the benefit of being better aware of available trials at the time of referral, thus allowing more of their patients to be enrolled. However, regardless of the referral source, educating referring physicians about the factors and characteristics that make patients ineligible may reduce the number of inappropriate referrals. Additionally, having a triage system to pre-screen referral records, such as prior therapy, laboratory results, concomitant medications, etc. may also help to ensure that more potentially eligible patients are seen in the phase I clinic.

As a retrospective study, our study has certain limitations. Although physicians' clinic notes are a useful and easily accessible source of information, they are hardly a complete record of all the complexities surrounding a patient's assessment or decision to undergo or decline a clinical trial. Certainly, in the case of 7 patients, it was unclear as to why they did not enter a trial, as well as in 10 patients in which no reason for declining trial entry was given. Additionally, 29 patients were lost to follow-up.

In examining the patterns of referral and enrollment in the phase I clinic over the past five years, our study provides future patients and physicians with better insight into the clinical realities underlying a referral to the phase I clinic. Our results identify factors and characteristics that hinder patient accrual, the knowledge of which may be helpful in defining referral guidelines and developing strategies to maximize patient recruitment.

## Conclusion

This study reviews the referral experience from the phase I clinic at the Princess Margaret Hospital, with focus on the barriers that influence eventual trial enrollment. Better selection of patients, appropriate education of referring physicians, and opening phase I trials with fewer restrictions on some criteria such as prior therapy may enhance their recruitment rates.

## Competing interests

The author(s) declare that they have no competing interests.

## Authors' contributions

JH participated in the study design, data abstraction, and manuscript preparation.

GRP participated in the study design, and performed the statistical analysis.

CN participated in the study design and coordination.

MM participated in the study design and in the management of study subjects reviewed in this manuscript.

EXC participated in the study design and in the management of study subjects reviewed in this manuscript.

AMO participated in the study design and in the management of study subjects reviewed in this manuscript.

LLS conceived of the study, participated in the study design and in the management of study subjects reviewed in this manuscript, and provided administrative support for the conduct of the study.

All authors read and approved the final manuscript.

## Pre-publication history

The pre-publication history for this paper can be accessed here:



## Figures and Tables

**Table 1:** Patient characteristics

**Age**	Mean (sd)	56.5 (11.1)
**Distance from PMH (km)**	Median (range) [IQR]	22.2 (0.6–1480.2) [12.7–42.7]

**Gender**	Male:Female	339:328 (50.8:49.2)

**Tumor site**	Breast	46 (6.9)
	GI colorectal	172 (25.8)
	GI non-colorectal	178 (26.7)
	Genitourinary	36 (5.4)
	Gynaecologic	74 (11.1)
	Head and neck	23 (3.5)
	Lung	57 (8.6)
	Sarcoma	8 (1.2)
	Skin and melanoma	11 (1.7)
	Unknown primary	42 (6.3)
	Multiple sites*	6 (0.9)
	Other**	14 (2.1)

**Number of prior regimens**	0	68 (10.2)
	1	210 (31.5)
	2	186 (27.9)
	3	116 (17.4)
	4	52 (7.8)
	5	24 (3.6)
	6+	11 (1.7)

**PS**	0	211 (31.6)
	1	318 (47.7)
	2	87 (13.0)
	3	49 (7.4)
	4	2 (0.3)

**Entered on trial**	Yes:No	197:470 (29.5:70.5)

**Referring physician**	PMH Oncologist	328 (49.2)
	External Oncologist	310 (46.5)
	Family Physician	12 (1.8)
	Other Physician	13 (2.0)
	Unknown	4 (0.6)

* Multiple sites were GI non-colorectal/gastric/lymphoma, GI non-colorectal/prostate, lung/breast, lung/esophageal, skin and melanoma/leukemia, unknown/prostate

** Others were adrenal [1], GI stromal [1], lymphoma [2], merkel cell [1] mesothelioma [3], mixed germ cell [1], neuroendocrine [2], pseudomyxoma [1], thyroid [1] and thymus [1]

**Table 2:** Breakdown of patient outcome by patient characteristics

		**N**	**Entered on Trial (% of Row Total)**	**Ineligible (% of Row Total)**	**Refused (% of Row Total)**	**Other Treatment Suggested First/No Spots Available/Initially Eligible but Deteriorated (% of Row Total)**	**Doing Well, Treat when Progress/Unclear/Lost (% of Row Total)**
**N**		667	29.5	24.7	14.4	24.9	6.5

**Age**	Mean (sd)^	667	56.6 (10.3)	57.3 (11.5)	57.8 (11.3)	54.3 (10.4)	58.4 (14.0)
	% 70+	75	21.3	30.7	17.3	16.0	14.7
	%<70	592	30.6	24.0	14.0	26.0	5.4

**Distance from PMH (km)**	Median [IQR]^	667	21.9 [12.6–38.2]	21.7 [14.2–42.7]	20.7 [12.6–35.5]	28.2 [12.6–57.6]	22.2 [12.7–59.5]

**Gender**	Male	339	30.1	23.9	12.7	26.0	7.4
	Female	328	29.0	25.6	16.2	23.8	5.5

**Tumor site**	Breast	46	23.9	39.1	15.2	19.6	2.2
	GI colorectal	172	23.8	25.0	14.0	30.2	7.0
	GI non-colorectal	178	33.7	22.5	16.3	20.8	6.7
	Genitourinary	36	30.6	27.8	13.9	19.4	8.3
	Gynaecologic	74	44.6	17.6	13.5	23.0	1.4
	Head and neck	23	56.5	4.4	13.0	17.4	8.7
	Lung	57	17.5	24.6	10.5	38.6	8.8
	Sarcoma	8	12.5	37.5	12.5	25.0	12.5
	Skin/melanoma	11	27.3	27.3	9.1	18.2	18.2
	Unknown primary	42	26.2	26.2	14.3	26.2	7.1
	Multiple sites	6	0.0	100.0	0.0	0.0	0.0
	Other	14	21.4	21.4	28.6	21.4	14.3

**Number of prior regimens**	0	68	47.1	20.6	14.7	14.7	2.9
	1	210	32.9	20.5	14.8	25.7	6.2
	2	186	24.2	25.3	14.5	26.9	9.1
	3+	203	25.1	30.1	13.8	25.6	5.4

**ECOG**	0	211	43.6	9.0	16.1	24.2	7.1
	1	318	28.6	17.3	16.0	29.6	8.5
	2	87	14.9	49.4	12.6	21.8	1.2
	3+	51	2.0	94.1	0.0	3.9	0.0

**Referring physician**	PMH Oncologists	328	38.4	21.3	15.9	21.3	3.1
	External Oncologists	310	19.0	29.4	12.6	29.0	10.0
	Others/Unknown	29	41.4	13.8	17.2	20.7	6.9

^ Entries in these rows are in units as listed
